# Round and flat zygomatic implants: effectiveness after a 3‑year follow‑up non‑interventional study

**DOI:** 10.1186/s40729-024-00548-9

**Published:** 2024-06-10

**Authors:** Carlos Aparicio, Waldemar D. Polido, Antoine Chehade, Marc Shenouda, Madalina Simon, Peter Simon, Bilal Al-Nawas

**Affiliations:** 1 Zygomatic Unit, Hepler Bone Clinic, ZAGA Center Barcelona, Roman Macaya, 22-24, 08022 Barcelona, Spain; 2 International Teaching Scholar, Indiana University School of Dentistry, Indianapolis, USA; 3 Oral and Maxillofacial Surgery, Indiana University School of Dentistry Indianapolis, USA. ZAGA Center, Indiana University, 1121 W. Michigan Street, Indianapolis, IN 46202 USA; 4 Seaforth Oral Surgery, ZAGA Center Montreal, 3550 côte des neiges, suite 170, Montreal, QC H3H 1V4 Canada; 5 ZAGA Center Stuttgart, All-On-4 Excellence Center, Kronprinzstraße 11, 70173 Stuttgart, Germany; 6 Department for Oral and Maxillofacial Surgery, Plastic Surgery, University Medical Center of the J. Gutenberg University Mainz, Augustusplatz 2, 55131 Mainz, Germany

**Keywords:** Zygomatic implants, ZAGA implants, ZAGA-flat, ZAGA-round, ZAGA, ORIS criteria, Zygomatic implant late complications

## Abstract

**Purpose:**

This non-interventional study investigates variations in the type and frequency of late complications linked to novel zygomatic implant designs, installed adhering to the Zygoma Anatomy-Guided Approach (ZAGA) concept, over an extended follow-up period of at least 3 years.

**Methods:**

Consecutive patients presenting indications for treatment with ZIs were treated according to ZAGA recommendations. Implants were immediately loaded. The ORIS success criteria for prosthetic offset, stability, sinus changes and soft-tissue status were used to evaluate the outcome.

**Results:**

Twenty patients were treated. Ten patients received two ZIs and regular implants; one received three ZIs plus regular implants, and nine received four ZIs. Fifty-nine ZIs were placed: thirty-six (61%) Straumann ZAGA-Flat implants and twenty-three (39%) Straumann ZAGA-Round implants. Four patients (20%) presented earlier sinus floor discontinuities. Fifteen patients (75%) had prior sinus opacities. Nineteen patients were followed for between 38 and 53 months (mean 46.5 months). One patient dropped out after 20 months. When comparing pre-surgical CBCT with post-surgical CBCT, 84.7% of the sites presented identical or less sinus opacity; nine locations (15%) showed decreased, and another nine increased (15%) post-surgical sinus opacity. Fifty-three ZIs (89.8%) maintained stable soft tissue. Six ZIs had recessions with no signs of infection. ZIs and prosthesis survival rate was 100%.

**Conclusions:**

The study highlights the effectiveness of ZAGA-based zygomatic implant rehabilitations using Round and Flat designs. Despite patient number constraints, minimal changes in the frequency of late complications from the 1-year follow-up were observed. 100% implant and prosthesis survival rate over a mean follow-up of 46.5 months is reported.

**Graphical Abstract:**

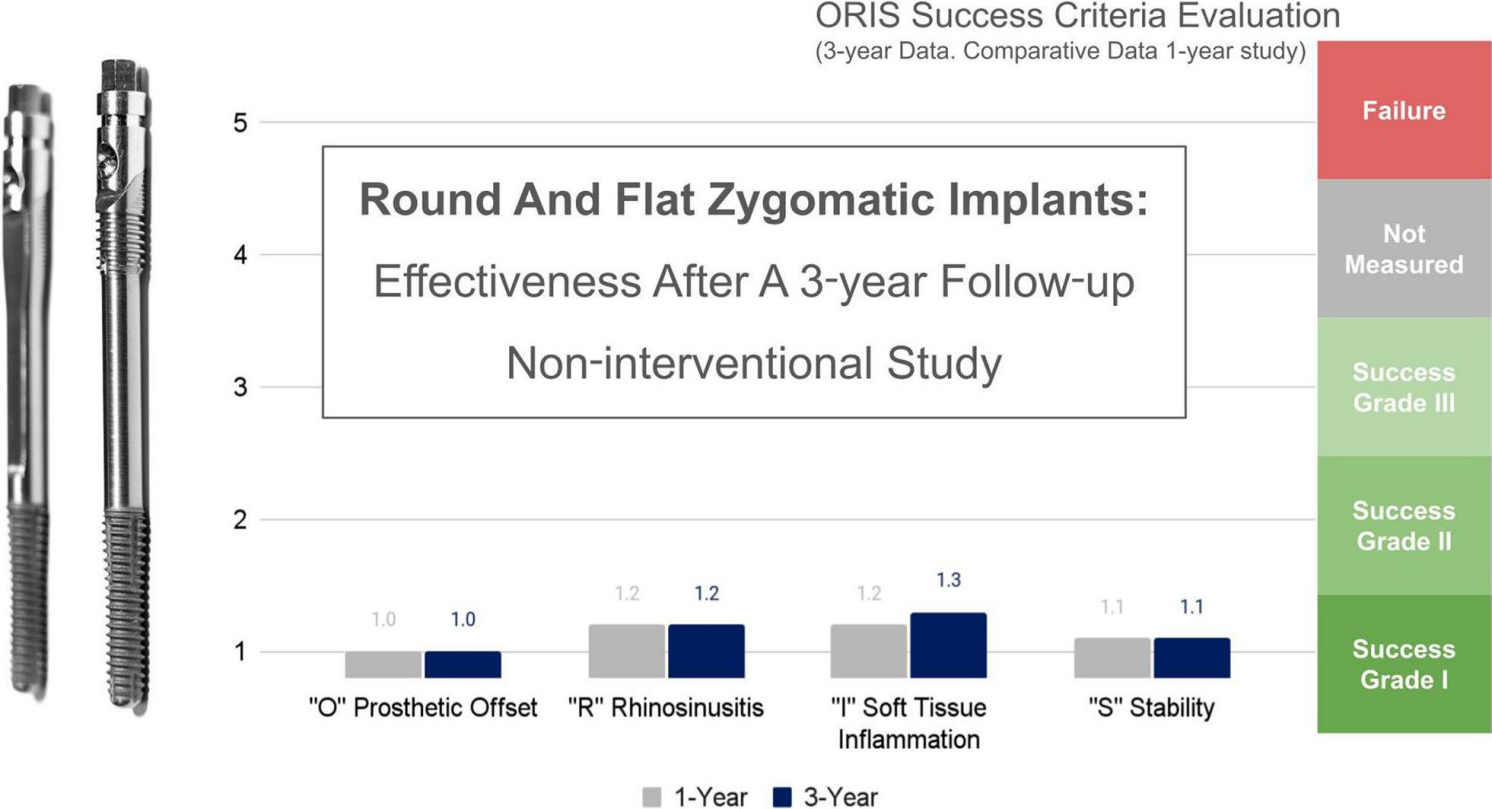

## Background

Since the 1990s, zygomatic implants (ZI) have been used to rehabilitate atrophic maxillae [1] and reconstruct maxillary defects [2]. They have demonstrated commendable survival rates [3–6] and emerged as an alternative to extensive bone grafting [7].

Despite their efficacy, surgical techniques for ZI’s placement have been consistently associated with perioperative complications, with additional issues surfacing in a delayed manner [7–9]. These complications have been linked not only to the surgical approach [9–12] but also to the macro and micro design of the implant itself [13, 14].

Implant placement techniques and designs have evolved over time in efforts to reduce complications. Stella and Warner introduced a technique to minimize osteotomy aggressiveness and enhance prosthetic emergence, although it did not prevent sinus complications [15, 16].

In 2003, Boyes-Varley implemented enhancements to the implant [17], elevating the platform correction to 55°, enhancing the prosthetic emergence. To mitigate bacterial leakage, Nobel Biocare refined the original Brånemark “zygomatic fixture” design by relocating the abutment fixation screw within the implant head and incorporating a textured surface (TiUnite). The new ZI was named Brånemark System Zygoma. Subsequently, they eliminated the threads in the body of the ZI, evolving it into the new “Nobel Zygoma” implant.

The extra sinus approach emerged to prevent late sinusitis, albeit with new complications such as soft tissue dehiscence and infection [18–20]. Subsequently, Chow [21] suggested extended osteotomy to reduce sinusitis risk. The Zygoma Anatomy-Guided Approach (ZAGA) [22, 23] provided a framework for surgeons to select the appropriate technique based on patient anatomy, enhancing predictability and reducing long-term complications [11, 12].

Recent developments include introducing two new zygomatic implant designs, ZAGA Round and ZAGA Flat, adaptable to various osteotomy patterns and implant trajectories [24]. These designs aim to fulfill three objectives within the ZAGA protocols: ensuring primary implant stability, minimizing the risk of dehiscence or soft tissue infection, and preserving zygomatic bone, maxillary wall, and alveolar bone remnants. Initial 1-year results using these implants following the ZAGA philosophy have been previously published [25].

This non-interventional study explores potential variations in the type or frequency of late complications associated with zygomatic implants with an extended follow-up of a minimum of 3 years. Recognizing that late complications may manifest at different intervals, we plan to present data up to 10 years post-loading, with this being the second of our planned publications.

## Material and methods

### Study population

This study is a prospective non-interventional investigation in which patients with indications for zygomatic implants voluntarily participated. The inclusion criteria used for treatment with zygomatic implants were anatomical and included partial or total maxillary edentulism; anterior, posterior or anterior and posterior maxillary atrophy, with or without discontinuities in the maxillary floor, implying the non-viability to place conventional implants without grafting procedures. Smokers were defined as individuals who smoked more than ten cigarettes per day. As this was a non-interventional study, no other specific inclusion or exclusion criteria were determined.

### Implant designs

**Fig. 1:**
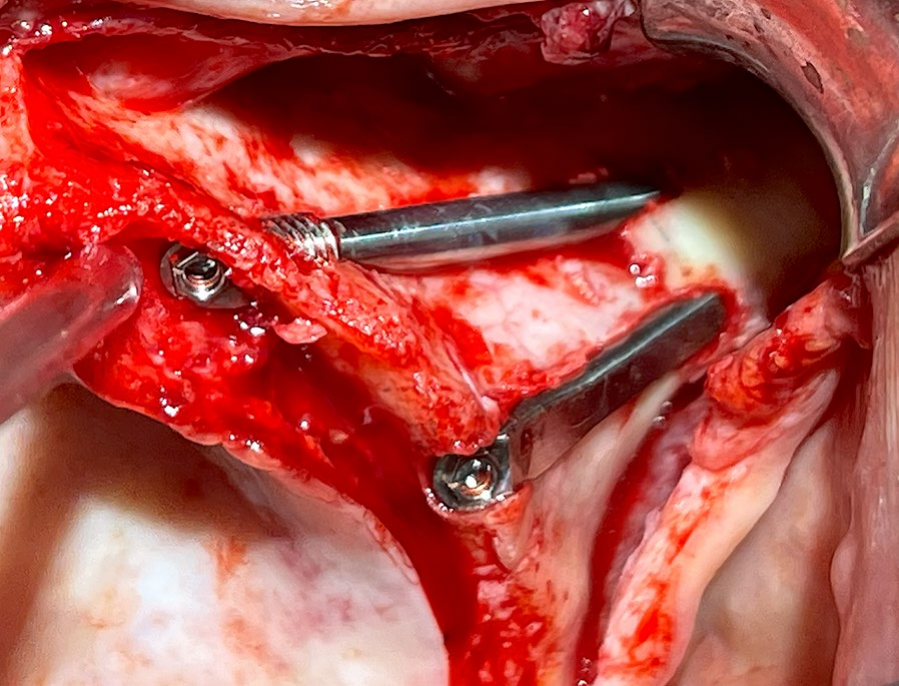
The clinical photograph shows two different Straumann ZAGA implants. In the anterior position, we can visualize the ZAGA Round model with a round section and macro-threads in its collar. In the premolar/molar position, we see the ZAGA Flat model. It has a circumferential arch section designed not to compress the soft tissue

The zygomatic implant system used (Straumann®, Switzerland) incorporates two distinct designs: the ZAGA™ Round, characterized by a circular section, and the ZAGA™ Flat, characterized by a circular segment section in its body and coronal region (Fig. 1). Both designs share a 55° directional correction integrated into their platform. The apical portion, similar in both implants, is tapered with a 2.6 mm apex and a 3.9 mm diameter body, threaded with a 0.8 mm thread pitch, and roughened. The body and the coronal region present a smooth machined surface. It should be noted that this implant system uses a fixation mount with the same diameter as the coronal part of the implant (4.3 mm). Immediate loading of all implants is recommended once good primary stability is obtained, along with adequate occlusal loading.

### Study protocol

**Fig. 2:**
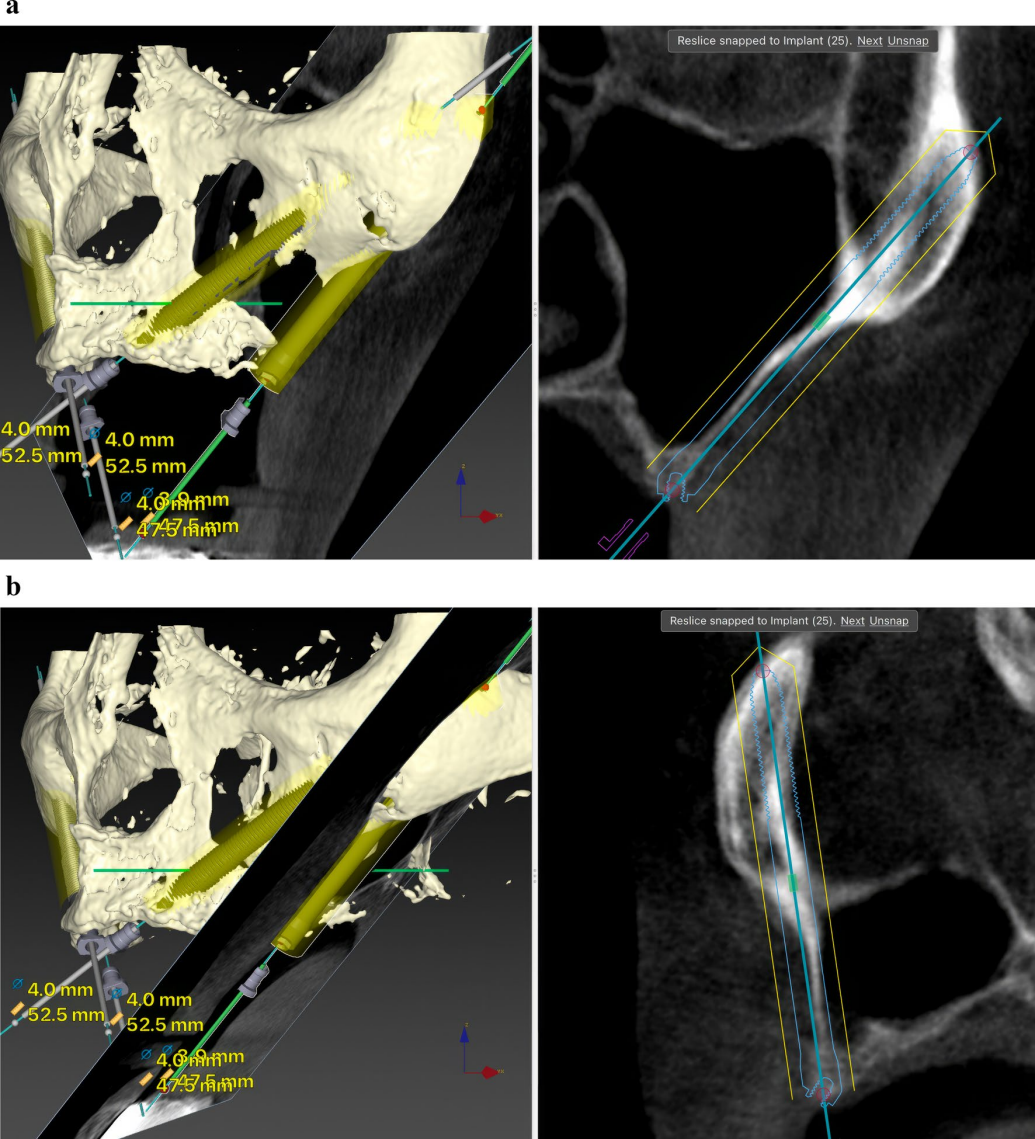
**a** Oblique CBCT slice, taken using DTX Studio Implant software, showing the planning and anatomy at the level of the second premolar/upper left first molar of the ZAGA Flat implant in Fig. 1. **b** The DTX Studio Implant software allows the rotation of the oblique plane attached to the implant. Thus, we can observe that the planning for the placement of the ZAGA Flat implant in Fig. 1 includes the partial use of the anterior wall of the temporal fossa. By increasing the number of cortices crossed by the implant, we will increase its primary stability

**Fig. 3:**
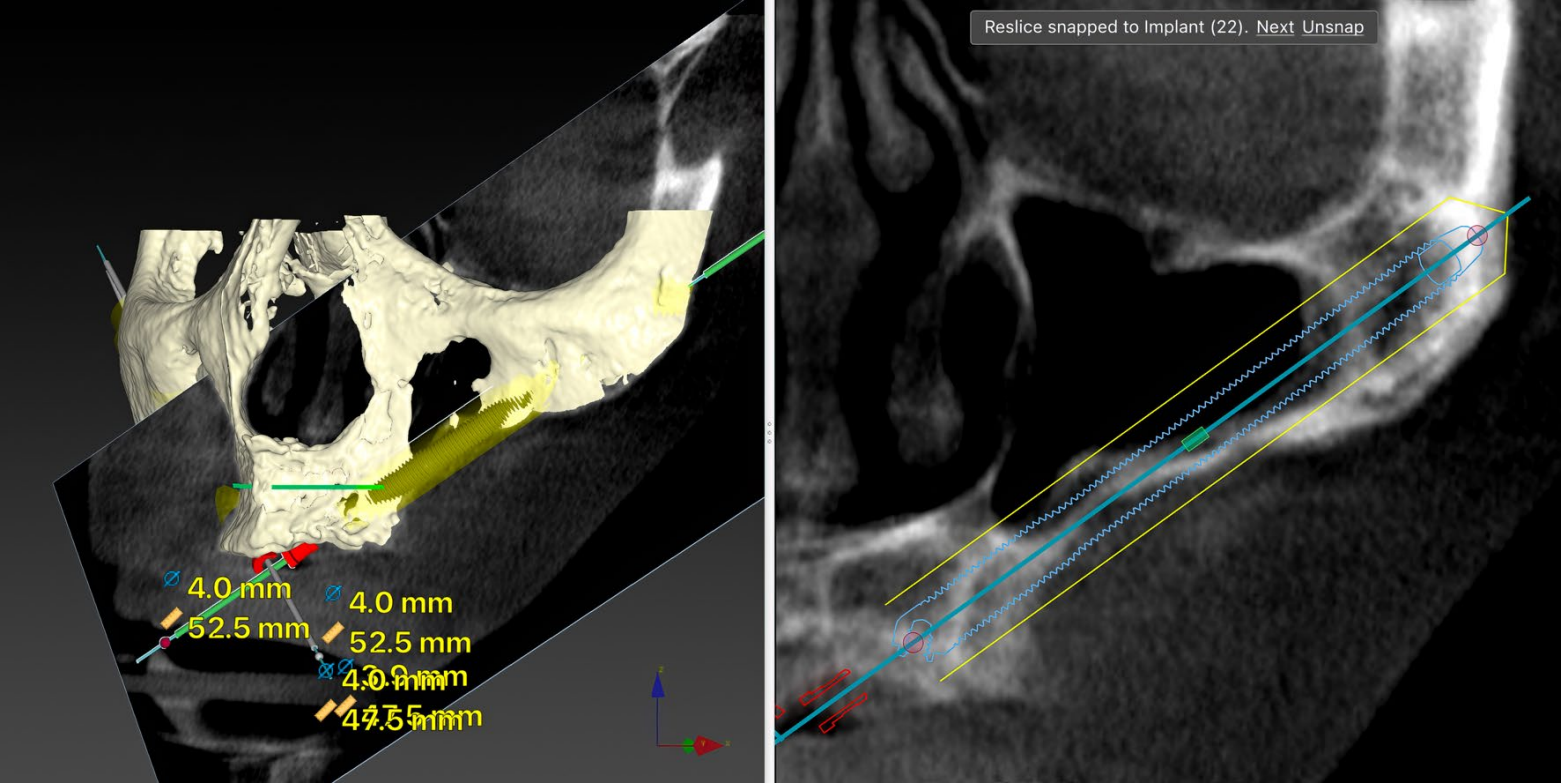
The oblique CBCT slice, taken using DTX Studio Implant software, shows the planning and anatomy at the level of the left lateral incisor of the ZAGA Round implant in Fig. 1. Although the patient moved during the acquisition, we declined to perform another CBCT

**Fig. 4:**
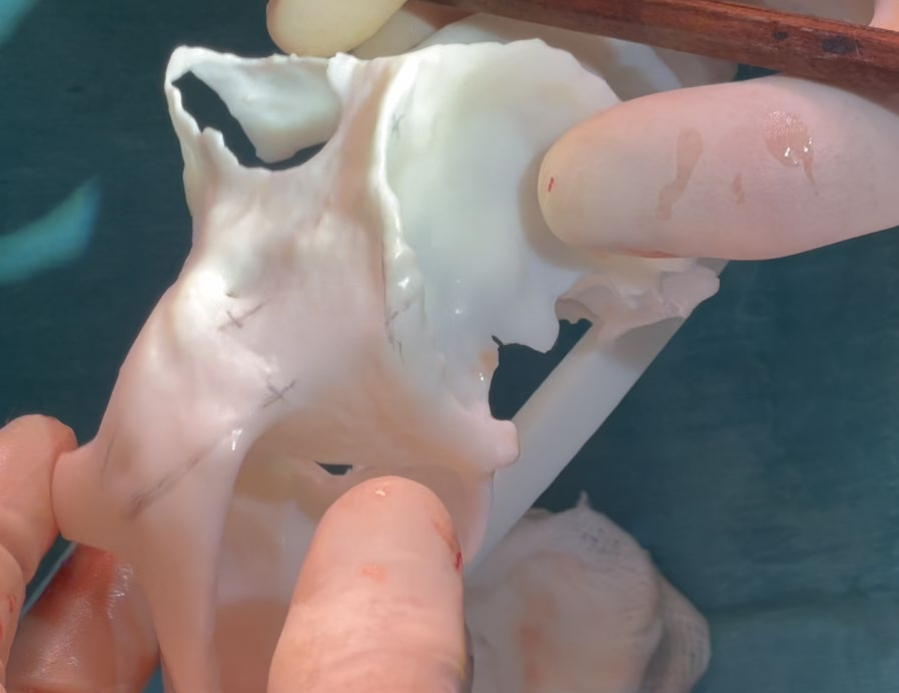
3D stereolithographic model obtained with manual segmentation used to familiarize ourselves with the patient’s anatomy and planning. Models provided by ZAGA Centers S.L., Barcelona, Spain

Before ZI placement, all patients underwent a thorough oral and radiological examination. After completing the medical history, an oral examination was performed with particular emphasis on oral opening, intermaxillary relationship, smile line, and mandibular dentition status. Patients diagnosed with periodontitis in the lower jaw, in which the affected teeth had to remain in the mouth, received preoperative periodontal treatment. The teeth to be extracted in the upper jaw also received periodontal treatment. In cases of extractions of posterior teeth in the upper jaw, a minimum period of 4 months was prescribed before the intervention to allow regeneration of the masticatory mucosa. The radiological examination included a maxillary cone beam computed tomography (CBCT). The minimum extent of the examined field included from the middle of the orbit to the alveolar ridge and both zygomatic arches. When the device’s field of view (FOV) allowed it, the frontal sinuses and the mandible were also included. This scan was essential for digital planning, facilitated by Nobel Biocare AG’s DTX Studio Implant software. Virtual implant osteotomies were planned using the ZAGA method that incorporates a single minimally invasive osteotomy (Figs. 2, 3), with objectives described by Aparicio et al. [23, 26, 27]. The DICOM images were manually segmented and transformed into STL files by an engineer (AS) to convert them into highly accurate stereolithography models (ZAGA Centers, Barcelona, Spain) where the planning could be completed, and a rehearsal surgery could be performed before the real one (Fig. 4).

The ZAGA minimally invasive zygomatic osteotomy goals were described extensively in the original 1-year follow-up article [25].

**Fig. 5:**
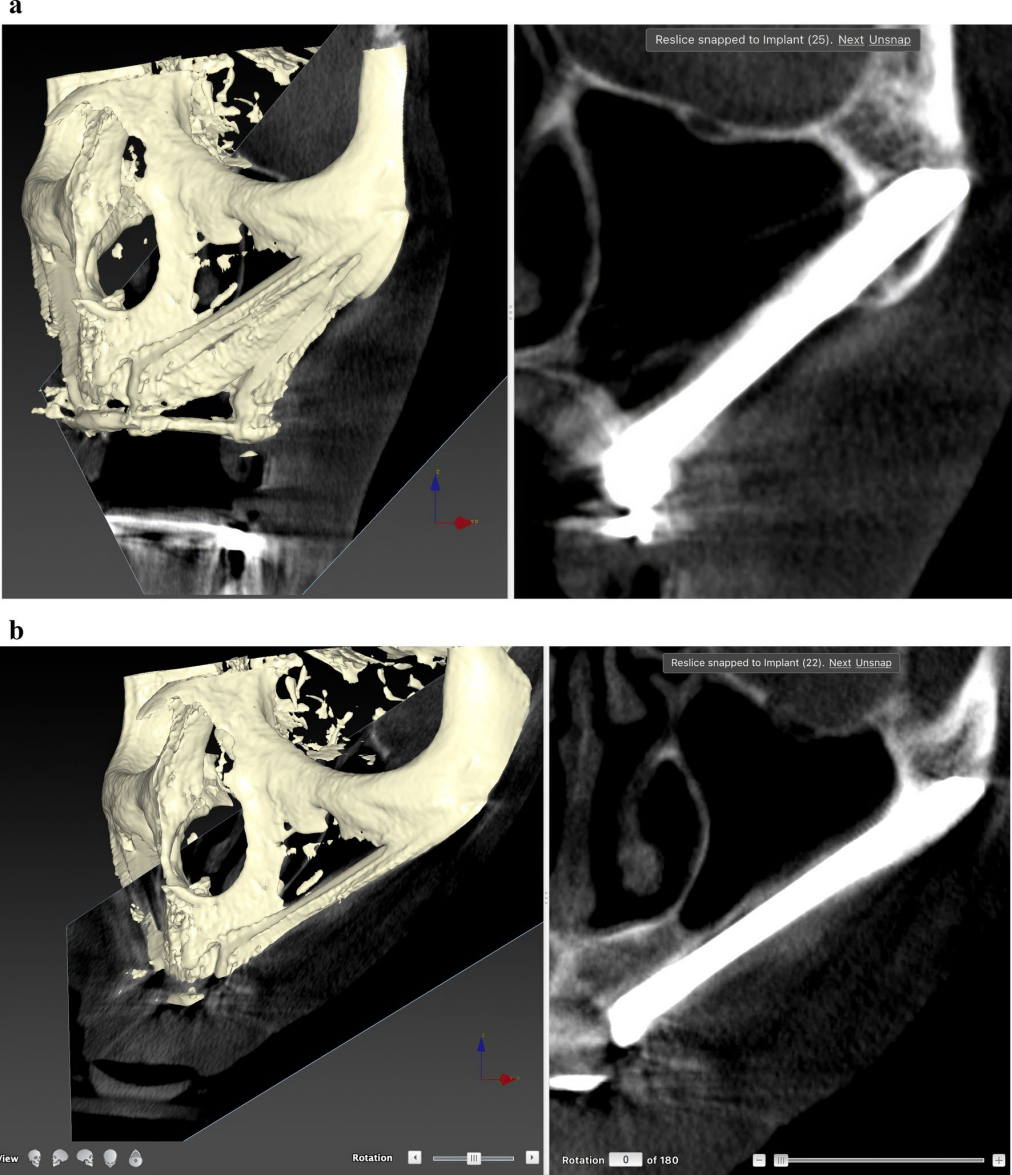
**a** Oblique CBCT slice taken in the control of the Straumann ZAGA Flat implant placed in the left posterior position in Fig. 1. The image, taken using DTX Studio Implant software, is to be compared with the one in Fig. 2a before the placement of the ZI. This will enable us to understand the condition of the implant and the possible variations in the adjacent structures. **b** Oblique CBCT slice taken in the control of the Straumann ZAGA Round implant placed in the left anterior position in Fig. 1. The image, taken using DTX Studio Implant software, is to be compared with the one in Fig. 3 before the placement of the ZI. This will enable us to understand the condition of the implant and the possible variations in the adjacent structures

The comprehensive care of the subjects encompassed a multifaceted approach, including diagnosis, surgical intervention, immediate prosthesis placement within 24 h, and systematic follow-up after implant loading. The routine follow-ups consisted of evaluations at 2 weeks and 4 months after immediate loading of the screw-retained provisional prostheses, supplemented by essential appointments for delivery of the definitive prostheses. Subsequently, a periodic follow-up control visit was diligently scheduled every 6 months. A new CBCT was taken after definitive prosthesis placement or at the first annual follow-up. After the first yearly check-up, new CBCT scans were taken at the clinician’s discretion (Fig. 5a, b). Prosthesis failure was defined as a prosthesis that has to be replaced for any reason.

Baseline assessments included meticulous documentation of medical and dental histories, laying the groundwork for a thorough understanding of each subject’s health profile. Throughout the study, vigilant recording of complications was maintained, providing valuable information on long-term outcomes and challenges faced by participants.

The intricacies of implant planning and placement were systematically documented, capturing critical details such as implant type, dimensions, and exact location. Parameters such as insertion torque were rigorously recorded to enhance the completeness of the study.

### Clinical protocol

The oral and radiological examination was decisive in choosing the type and position of the incision in each patient according to the principles of individualization [26, 27]. The flap always included the possibility of placing a specially designed retractor in the angle formed by the frontal and temporal apophyses of the zygomatic bone (ZAGA Kit, Salvin Dental Specialties). This was followed by the detachment of the periosteum on the posterior maxillary wall to prepare an eventual utilization of the space of the temporal fossa, increasing the number of cortices crossed. The positioning of the implant’s coronal aspect was significantly influenced by both the size and structure of the alveolar/basal process, as well as the curvature of the anterior maxillary wall. If the bone structure around the nasal/sinus floor was judged adequate to accommodate the implant neck (at least 4 mm in height and 6 mm in width), the implant was positioned using a circular osteotomy resembling a tunnel shape (Fig. 3). In cases where a tunnel osteotomy was selected for ZAGA Type 1 anatomy, deliberate perforation of the sinus membrane occurred during the final stages of completing the antrostomy [15, 18]. We also applied this tunnel osteotomy approach in anatomical scenarios categorized as ZAGA type 3, characterized by a concave maxillary wall and remnants of alveolar bone arranged in a buccally inclined triangular pattern. In these cases, the antrostomy was placed far from the critical zone (Figs. 1, 3, 5b). The objective of employing the tunnel osteotomy technique was to achieve a durable seal, promoting osseointegration at the implant neck level through: (1) the stability of a zygomatic implant, (2) an appropriately threaded implant neck section ensuring proper distribution of masticatory forces, (3) sufficient surrounding bone at the neck, and (4) attachment to a rigid prosthesis. The tunnel type of osteotomy is normally used in ZAGA types 0, 1, and 3 and, by definition, has an alveolar entry of circular section; thus, the Straumann® ZAGA™ Round zygomatic implant featuring machined circular section and threads in the neck was chosen for these cases (Fig. 1).

If insufficient residual bone structure was observed at the crestal level (less than 4 mm in thickness), the coronal osteotomy was adjusted to a buccal position (Figs. 1, 2, 5a). The implant beds were designed to host both the neck and, ideally, the body of the implant within the alveolar bone and maxillary wall, while ensuring the preservation of sinus lining integrity at the coronal level. The antrostomy was performed as far away as possible from the critical zone in these cases. This particular osteotomy technique, characterized by encountering lateral walls and floor but no roof, is referred to as ‘channel osteotomy’. In these cases, the Straumann® ZAGA™ Flat design was chosen, which has a circular segment section to fit the channel, aiming at reducing vascular compression of the soft tissues (Fig. 1).

**Fig. 6:**
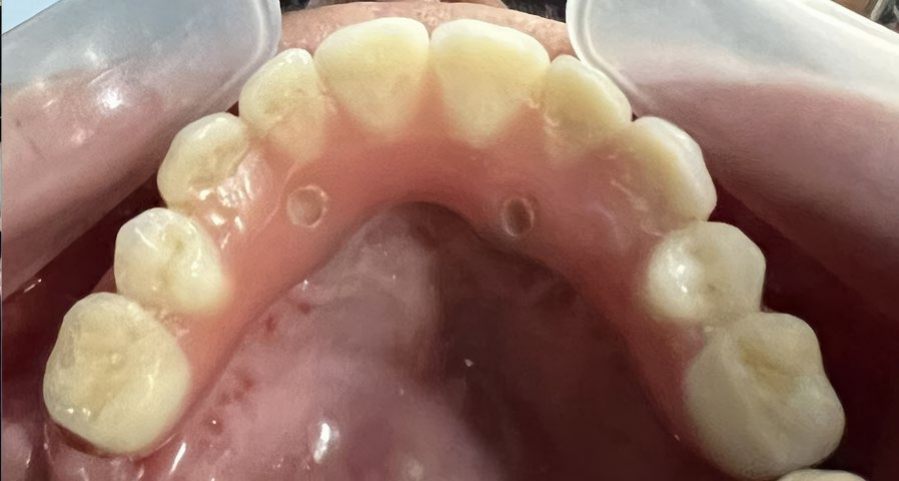
Immediate prosthesis placed on the implants in Fig. 1

Following implant surgery, an immediate screw-retained provisional prosthesis was universally placed in all patients, based on the individualized treatment plan determined by each center (Fig. 6). We strongly advised a non-smoking period of at least 2 weeks, along with adhering to a soft diet for 3 months. Following a period of 4–6 months, a more comprehensive screw-retained prosthesis was provided. One patient remained with reinforced provisional prostheses. If necessary, the final prostheses included an added cantilever until the completion of twelve teeth on the upper arch.

### Outcome measures

The outcome of the combination of the ZAGA protocols together with the new Straumann® Zygomatic Implant System was evaluated using the ORIS criteria for a systematic report of a zygomatic implant-related rehabilitation [28, 29].

**Fig. 7:**
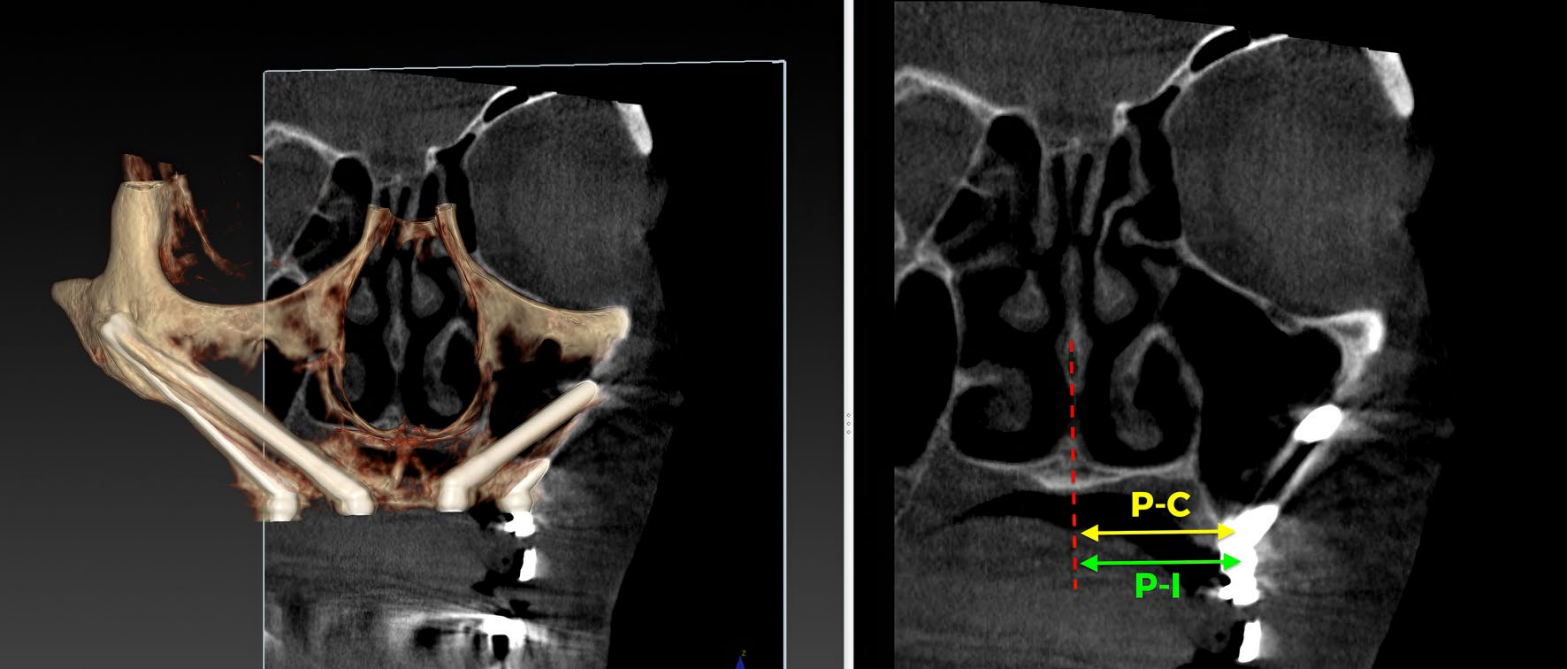
Using the DTX Studio Implant, the left 3D image is cut in an orthoradial plane to the emergence of the ZAGA Flat implant in Fig. 1. On the right side of the illustration, we visualize that the red dashed line divides the palate in two. In yellow, the P–C segment indicates the distance from the center of the palate (P) to the center of the ridge (C). In green, the P-I segment indicates the distance from the center of the palate to the center of the implant platform (I). The subtraction of both segments will indicate the measurement and type of offset, if any

**Fig. 8:**
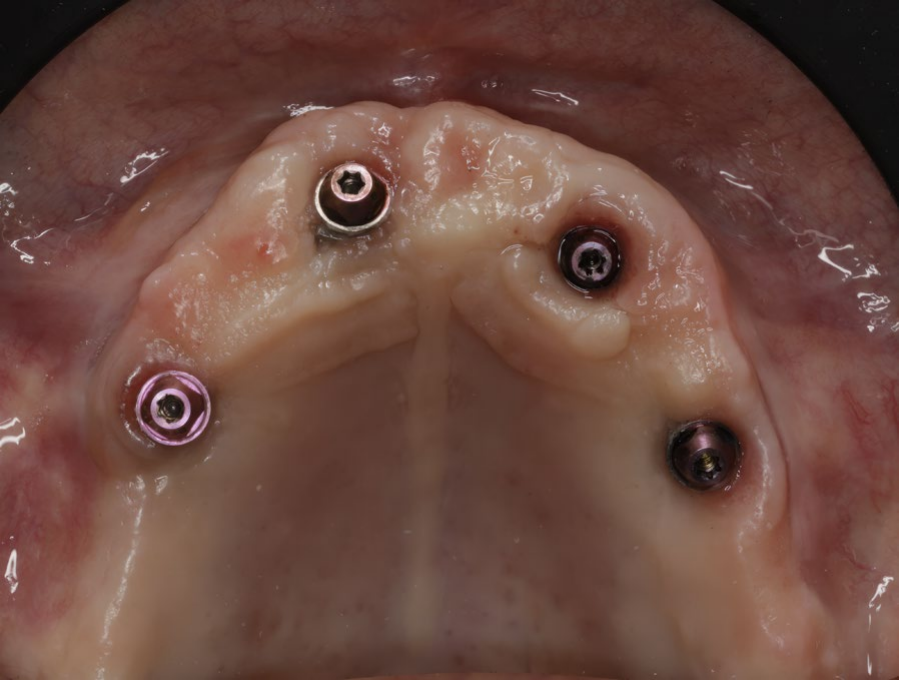
Status of soft tissues around Straumann abutments 50 months after surgery for zygomatic implant placement

The outcome evaluation followed four specific and objective criteria as used in the 1-year follow-up study [25]. Briefly, the criteria included:

- Offset evaluation of prosthetic success based on final positioning of the zygomatic implant compared to the center of the alveolar crest (Fig. 7).
- Rhino-sinusal evaluation (Figs. 2, 3, 5).
- Soft tissue infection/inflammation evaluation (Fig. 8).
- Stability evaluation.

Each criterion was assessed using the ORIS levels from Level I (success with optimal conditions) to Level V (failure).

Based on these criteria, one of five possible conditions is assigned to each implant. Following the ORIS criteria [28], two independent researchers (B Al-N and WP) from separate centers, blinded to the study, analyzed the radiological outcomes by comparing pre-operative and post-operative CBCT images. The analysis was simple and consisted of comparing the screenshot with the virtual planning of the implant with screenshots showing the actual implant already placed in the planned site. If the sinus opacity matched or was lower, it was deemed negative; any increase indicated a positive result. Both evaluators reached the same conclusions in their assessments. Clinical pictures of the mucosal status, implant positioning, and occlusal prosthesis view were analyzed by AC, MSh, PS, and MS in an anonymized manner. Implant and prosthesis survival rates, other complications, and patient satisfaction after 3 years of treatment were the study’s secondary endpoints.

## Results

### Patients

Twenty consecutive patients with indications for zygoma-related rehabilitation underwent treatment. The initial zygomatic surgery was conducted in June 2019, with the final procedure completed in October 2020. The results of their 1-year follow-up were previously published [25]. Ten patients were provided with two zygomatic implants in addition to standard anterior implants, while nine patients received four zygomatic implants. One patient received three zygomatic implants along with two regular implants on the anterior maxilla. Altogether, 59 zygomatic implants were inserted, comprising 36 Straumann® ZAGA-Flat and 23 Straumann® ZAGA-Round designs.

Among the subjects, seven individuals (35%) were identified as smokers. Four patients (20%) exhibited prior disruptions in the sinus-nasal floor. A total of fifteen patients (with 26 sites) showed previous opacities in the maxillary sinus. Among them, one patient (P-6) presented two affected sites within the same maxillary sinus, significantly impacting ostium patency without eliciting clinical symptoms. With the exception of four implants within the same patient (P-5), all implants achieved an insertion torque of over 45 Ncm. All patients received an immediate screw-retained prosthesis within 48 h. The definitive prosthesis usually included a bilateral posterior extension equivalent to the size of the first molar. In 19 patients, the final prosthesis was placed 4 to 6 months after loading.

One patient (P-2) disappeared from the study wearing the initial-reinforced prosthesis. Her last follow-up was performed 20 months after loading and included a CBCT, individual examination of implant stability, and soft tissue status registration. A patient (P-7) received two zygomatic implants in positions 13 and 22 and 3 regular implants in positions 16, 24, and 26. She lost the regular implants 24 and 26, so the prosthesis had to be modified by shortening the left side. She suffered a severe traffic accident, and in the trauma study, she was diagnosed with cancer in the right lung. As soon as possible, she will be scheduled for a second zygomatic implant on the left side. A patient (P-18) came to the 3-year check-up with movement in the prosthesis. He had fractured an abutment, which was replaced. Two patients (P-10 and P-15) experienced two and three episodes of oro facial swelling without radiological signs of sinus occupancy. Both were successfully treated with antibiotherapy. Once treated, 19 patients were followed for between 38 and 53 months (mean follow-up 46.5 months). The zygomatic implant survival rate was 100%. The prosthesis survival rate was 100%.

### ORIS level

The results of the ORIS measurements are shown in Table 1 and are detailed below.

**Table 1:** Outcome of the ORIS evaluation 3 years after the treatment

ORIS dimension	Score
“O” prosthetic offset	Level I for all implants
“R” rhinosinusitis status	Level I for 50 sites (15 patients *) Level II for 8 sites (4 patients) Level III for 1 site (1 patient)
“I” soft tissue stability/infection	Level I for 53 sites (15 patients) Level II for 5 patients 6 sites (5 patients)
“S” implant stability	Level I for 58 implants Level III for 1 implant

(*)9 sites in 7 patients presented more radiolucent sinuses in the last radiological examination after zygomatic implant placement than in the pre-surgical examination

#### Prostheses offset (the “O” criterion)

**Fig. 9:**
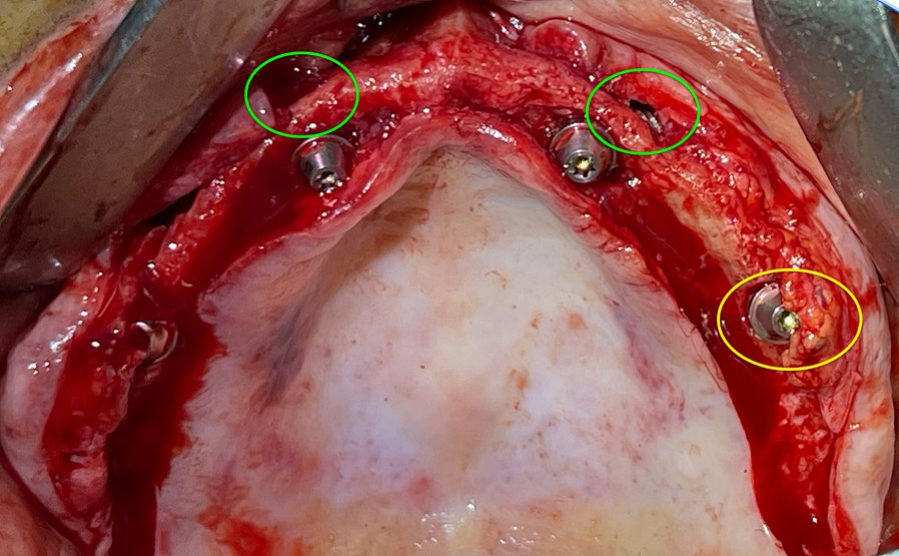
The occlusal photograph shows the final position of the implants with their abutments of the case illustrated in Fig. 1 and subsequent ones. The two Straumann ZAGA Round implants, placed in the anterior position, typically appear on the palatal side of the ridge (green circles). The platforms of the Straumann ZAGA Flat implants in posterior positions are placed in the middle of the ridge (yellow circle)

The assessment of the emergence of the implant head resulted in a level “I” for all implants. Consistently, the center of the implant head was positioned at the residual alveolar crest with the flat design or at a maximum of 4 mm palatal with the round design (Fig. 9).

#### Rhino-sinus condition (the “R” criterion)

Fifty sites (84.7%) showed either the same or increased sinus transparency when comparing pre-surgical with post-surgical CBCT scans: 15 patients showed negative (−) Modified Lund Mackay (M-LM) test; ORIS level 1.

Eight sites in 4 patients (13.5%) displayed a light increased sinus opacity that did not affect ostium patency and had no clinical significance ORIS level 2. In one instance, observed in a single patient (1.7%), there was a notable increase in opacity at a specific site, yet without compromising ostium patency, classified as ORIS level 3. In total five patients exhibited a positive result on the Modified Lund Mackay scale.

**Fig. 10:**
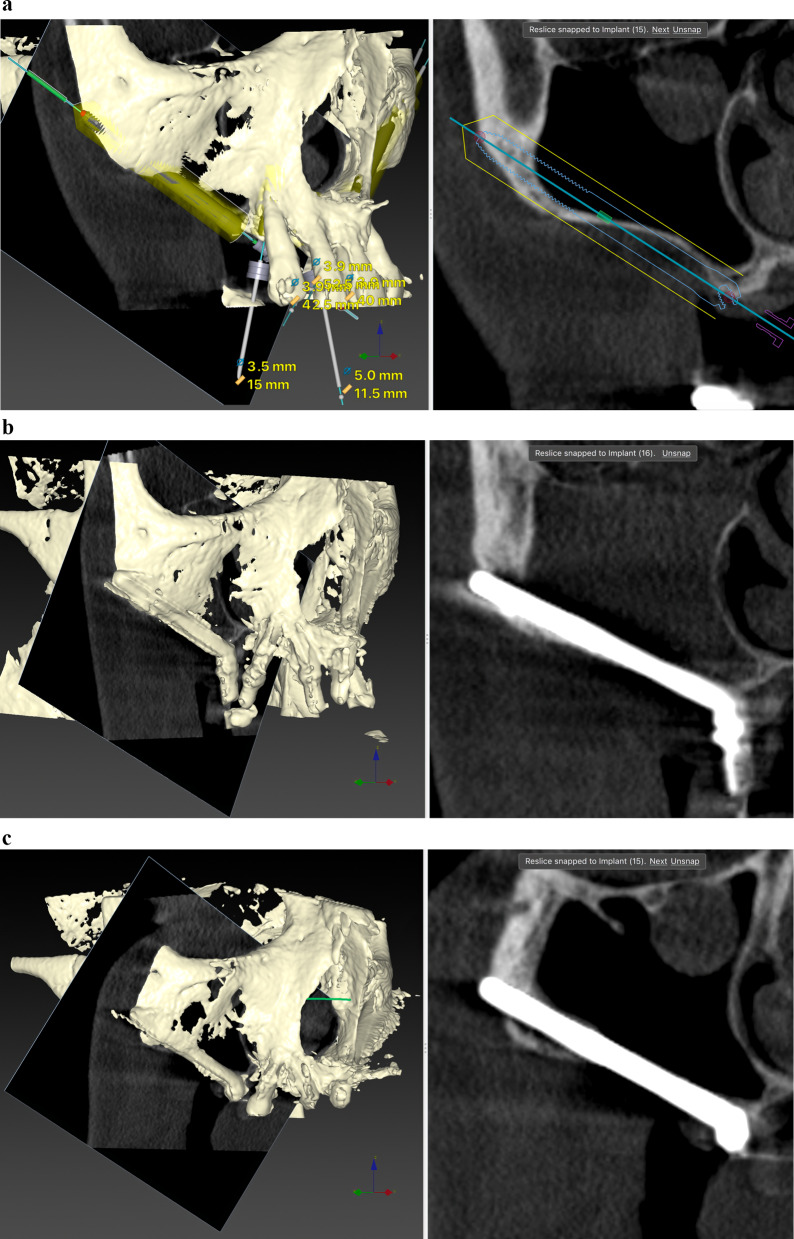

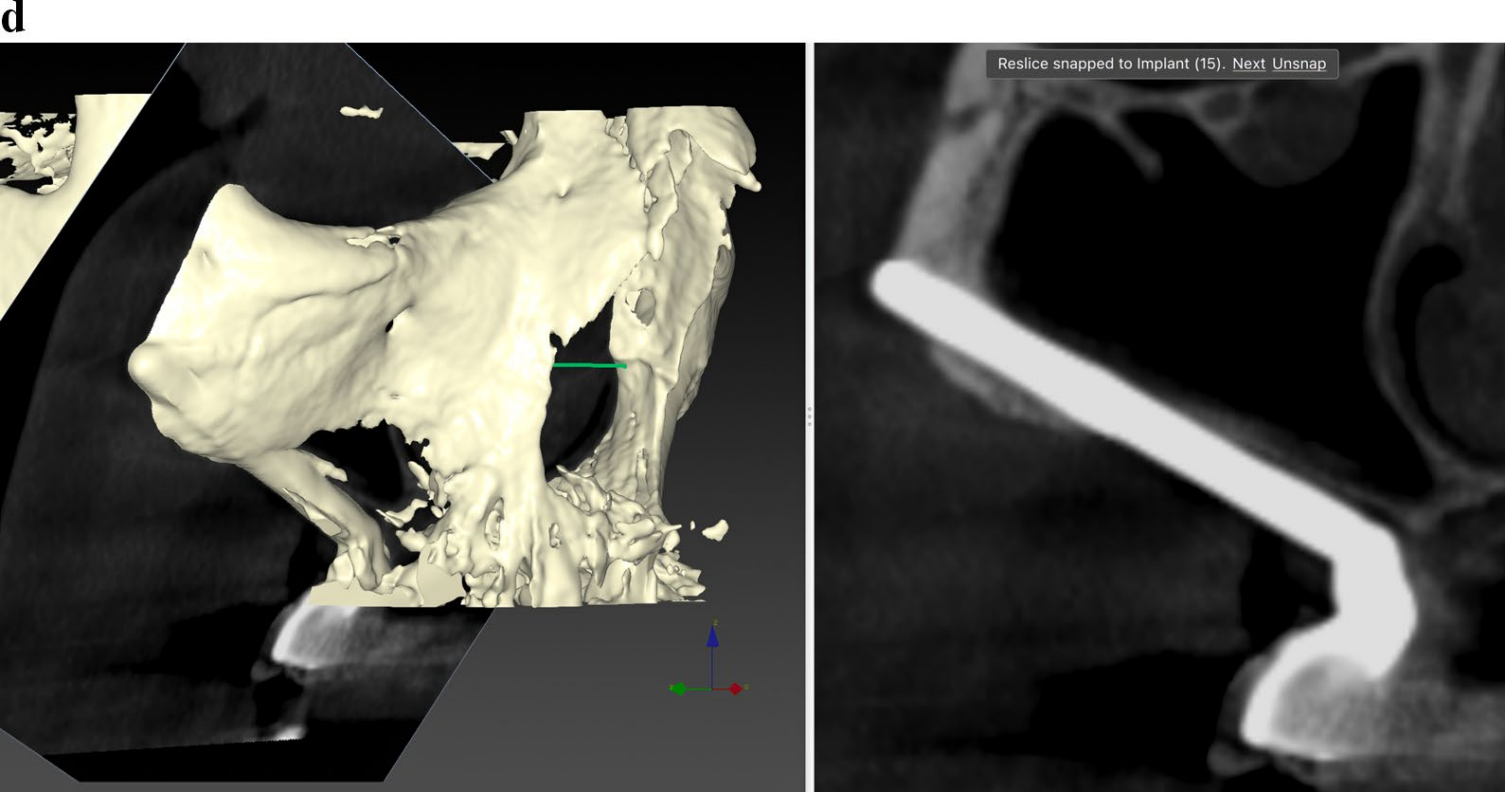
**a** Oblique CBCT slice taken on November 11th 2020. DTX Studio Implant software was used to plan the implant trajectory using the ZAGA Channel approach. **b** December 11th 2020. One week after surgery, planned in **a**, the patient presents with acute symptoms of rhinosinusitis. The oblique CBCT slice using DTX Studio Implant software shows complete opacity of the right maxillary sinus. **c** March 3rd 2021. Three months after surgery. After medical treatment, the clinician’s symptoms disappear, and when we compare the image of this figure with the one in Fig. 11a, we may verify that the M-LM is negative. **d** The DTX Studio Implant software allows us to superimpose a virtual implant on the real one and analyze it. The new CBCT was taken on December 22, 2023, 3 years after the surgery. When we compare it with the CBCT before surgery, we verify that the M-LM is negative

One patient exhibited clinical and radiological manifestations of acute rhinosinusitis 1 week post-surgery, which subsequently resolved following standard medical intervention (Fig. 10). New CBCT taken at 3 years and 2 months confirms the negativity of the M-LM test (Fig. 10d).

**Fig. 11:**
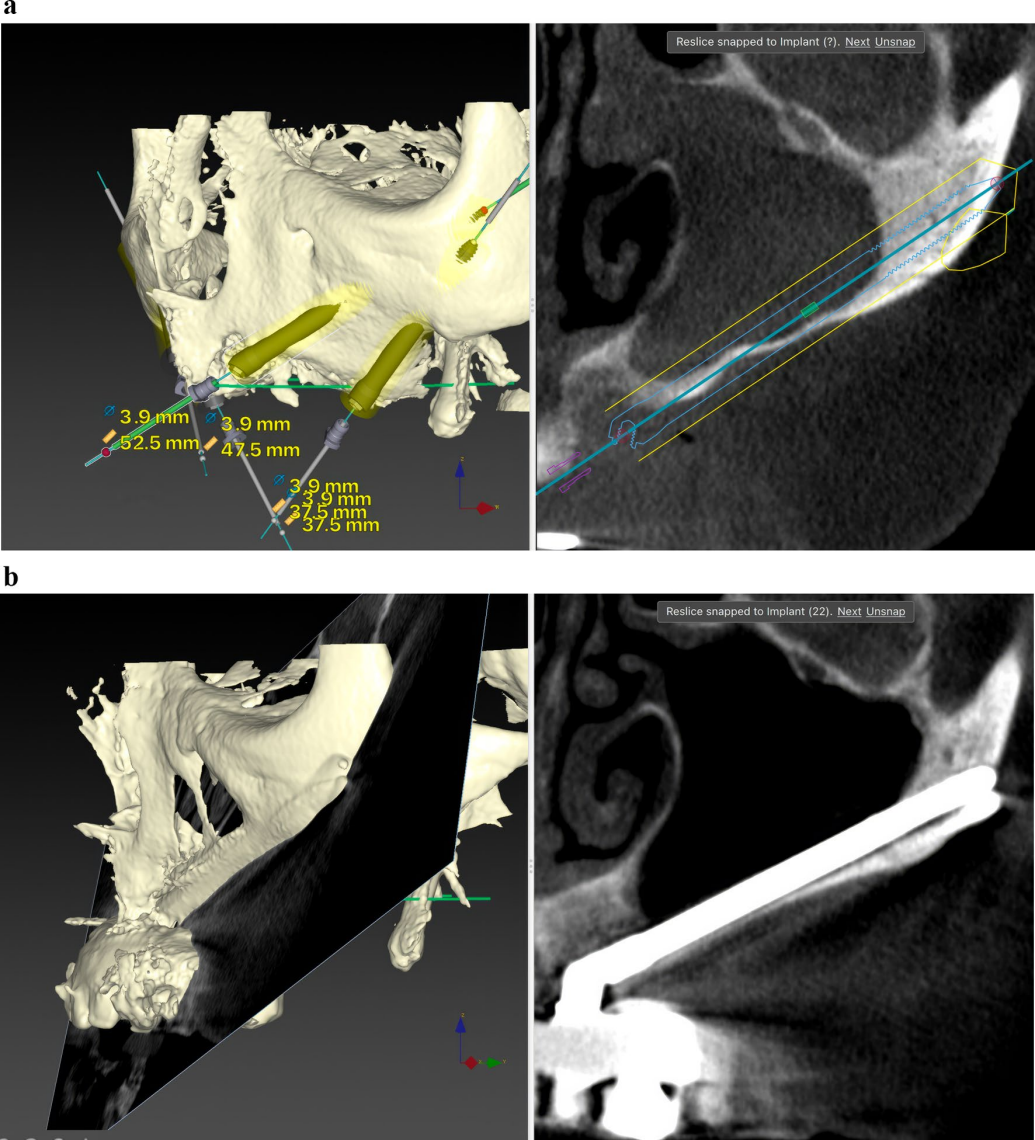
**a** This heavy smoker patient (P6) presented, as a consequence of implant failure and previous augmentation surgeries, multiple bony discontinuities in the sinus and nasal floor on radiological examination. As observed in the oblique CBCT slice taken in the direction of the left anterior implant, both the maxillary sinuses and the ostium are opaque. The patient had no clinical symptoms. **b** The same patient (P6) in **a** presented this CBCT image 18 months after surgery performed according to the recommendations of the ZAGA protocol and using a Straumann ZAGA Round implant. The authors need to determine the reasons for the improved patency at both the sinuses and the ostium. Note also the spontaneous healing of the initial osseous defect at the level of the left incisors

**Fig. 12:**
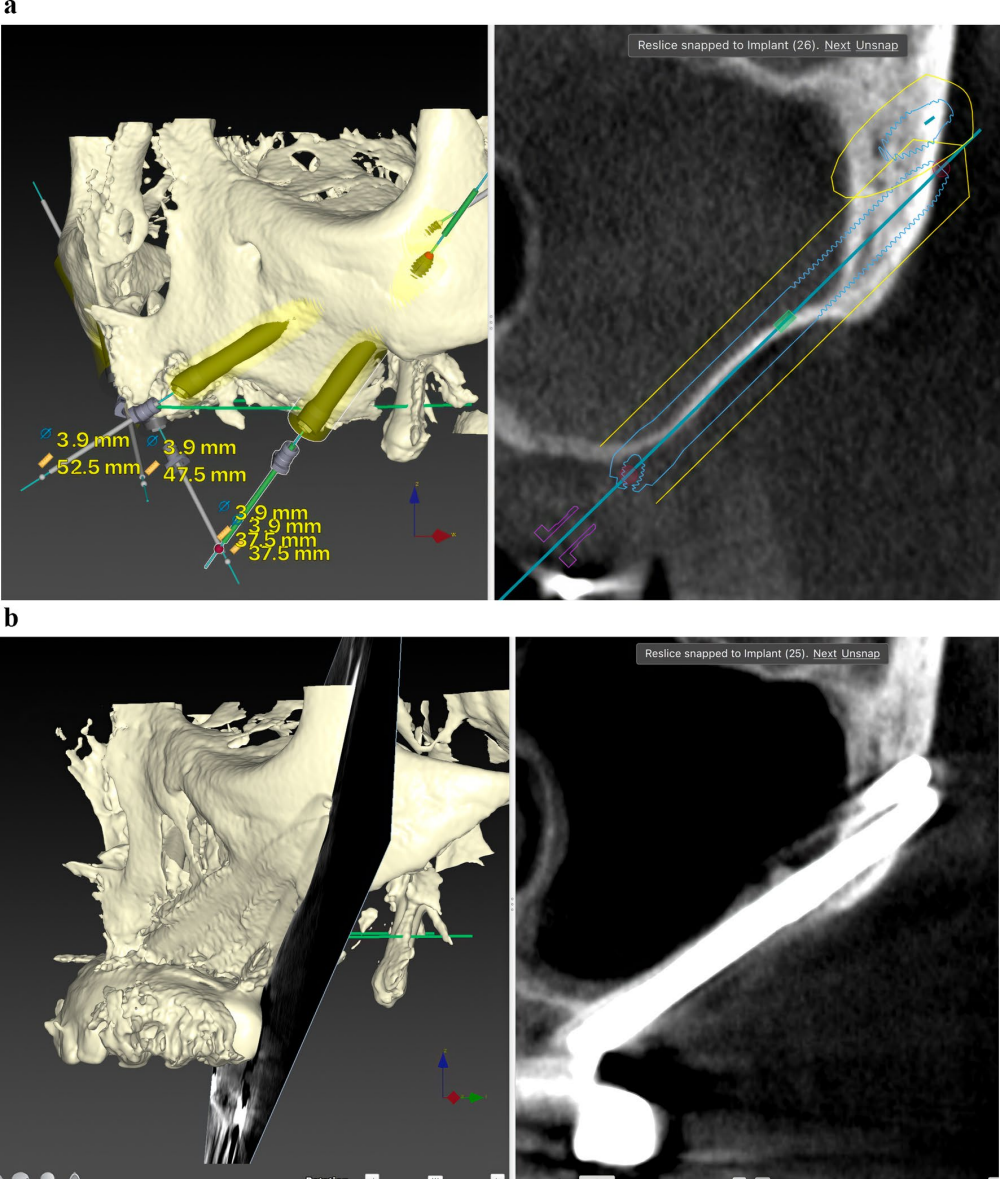
**a** The oblique CBCT slice shows the left posterior sector with a ZAGA channel osteotomy planning. As in the anterior sector, the maxillary sinus and the ostium are opaque. **b** The same patient (P6) in **a** presented this CBCT image showing sinus patency 18 months after a zygoma surgery performed according to the recommendations of the ZAGA protocol and using a Straumann ZAGA Flat implant

In seven patients (15%), nine locations demonstrated a reduction or absence of opacity when comparing pre-surgical CBCT scans to post-surgical CBCT scans obtained at the conclusion of the radiological assessment (Figs. 11, 12).

The Lanza Kennedy clinical test was negative at the final control in all patients. The ORIS evaluation of the Rhino-sinus condition led to a success level “I” in 14 patients, a success level “II” in 4 patients, a success level “III” in 1 patient, and a success level “IV” in 1 patient.

#### Soft tissue stability/infection (the “I” criterion)

**Fig. 13:**
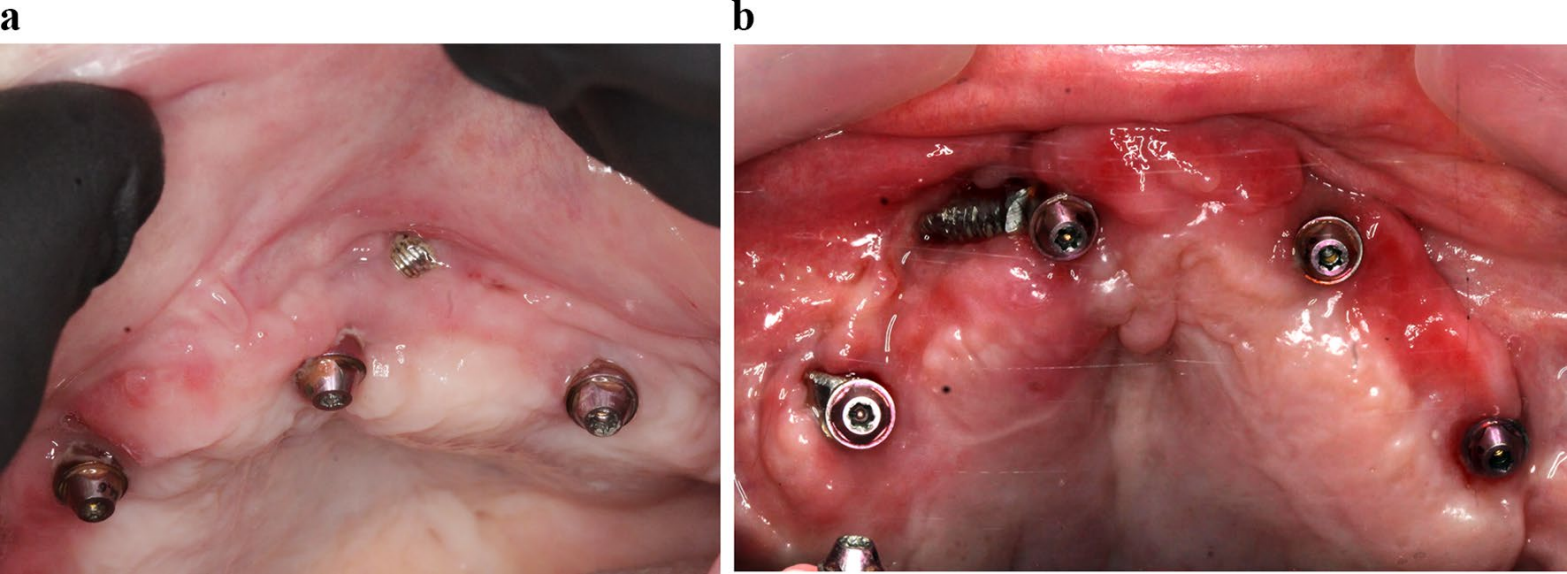
**a** The clinical photograph shows a soft tissue defect in the window pattern that appeared in this patient 2 years after surgery. Soft tissue color is normal. **b** To close the window in **a**, surgery was performed by mobilizing soft tissues and partially removing the threads from the collar of the implant. The result was a wider defect. After 3 years, it remains stable

Soft tissue stability was observed around fifty-three implants (89.8%), remaining unchanged. Six implants showed non-inflammatory soft tissue alterations (10.2%). Among these, non-inflamed soft tissue recession and inadequate prosthesis design were noted in one patient with two flat design implants. One round design implant placed in a heavy smoker showed a mucosal fenestration without inflammation in control at 18 months. The fenestration was surgically closed. However, 6 months later, a stable recession without inflammation appeared (Fig. 13). Another two patients showed a stable exposure of a flat implant 31 months after placement.

The assessment of soft tissue stability resulted in a success level of “I” for fifteen patients and a success level of “II” for five patients.

#### Implant stability (the “S” criterion)

Fifty-eight implants were evaluated as stable level I. One implant in an extremely atrophic maxilla and zygoma showed slight intrusional movement but no pain or rotational motion. Fifty-one months after the surgery this implant was still in function.

The assessment of implant stability yielded a success level of “I” for 19 patients and a success level of “III” for one patient. The results of the ORIS evaluation after 3 years are summarized in Table 1.

## Discussion

This prospective non-interventional clinical study aimed to evaluate the long-term clinical outcomes of 20 patients with severe maxillary atrophy. These patients completed at least 3 years of rehabilitation using fixed prostheses anchored to zygomatic implants (ZI). These implants, referred to as “site-specific implants” due to their versatility with different osteotomy techniques, were chosen and positioned in accordance with the Zygomatic Anatomy-Guided Approach (ZAGA) concept.

Building on an earlier 1-year follow-up study with the same cohort, this investigation delves into the evolution of treatment outcomes over time. Given the propensity for late complications in these types of procedures, this extended follow-up period provides valuable information on the durability and efficacy of the intervention.

The unique characteristics of zygomatic implants and their distinct anatomical context necessitate specialized evaluation criteria separate from those used for conventional implants. Specific criteria called ORIS have been introduced [28] to assess the outcomes of zygomatic implant-supported rehabilitations to address this need. These criteria enable clinicians to conduct standardized follow-ups, employing strict benchmarks tailored to the intricacies of zygomatic implant treatment. In contrast to traditional implant evaluations, the ORIS criteria prioritize critical factors such as prosthetic results, maxillary sinus integrity, peri-implant soft tissue condition, and stability of zygomatic implants. By utilizing ORIS indicators, practitioners can consistently and objectively evaluate zygomatic implant outcomes, facilitating comparability across reports and enhancing the quality of research in this field.

The study findings indicate that the surgical procedure and postoperative healing progressed smoothly, with no notable issues arising with any implants. Only one patient experienced immediate postoperative sinusitis, which was effectively treated with medical intervention and resolved both clinically and radiologically (see Fig. 10). Notably, the survival rates for both the zygomatic implants and the prostheses reached 100%, aligning with findings from prior research. A recent systematic review [5] underscores this consistency, reporting cumulative success rates of zygomatic implants for severe maxillary atrophy treatment: 98.5% at less than 1 year, 97.5% between 1 and 3 years, 96.8% between 3 and 5 years, and 96.1% after 5 years. The primary complications noted across studies include soft tissue dehiscence, rhinosinusitis, and prosthetic failures.

The study employs the Lanza–Kennedy clinical task force [30] and Lund Mackay modified radiological scoring system [28, 29] as standardized methodologies for categorrizing sinus status. Significantly, fifteen patients (involving 26 sites) had previous instances of maxillary sinus opacities, with one patient experiencing opacity in two sites severely impacting ostium patency, and four patients (20%) presenting previous discontinuities in the sinus-nasal floor. A comparison between pre- and post-surgical CBCT scans revealed that 84.7% of sites demonstrated either unchanged or increased sinus transparency. Conversely, five sites across various patients exhibited a minor increase in opacity without clinical relevance. The authors did not provide an explanation for the reduction or absence of opacity observed in nine sites among seven patients with opaque or partially opaque sinuses before surgery (Figs. 11 and 12). According to the ZAGA concept and the use of site-specific ZAGA implant designs, this phenomenon could be related to the anatomically-guided implant placement method. Still, it is outside the scope of the study to demonstrate this. Further investigation in a more extended follow-up setting is warranted to elucidate this observation fully.

Periodic evaluation of the soft tissues is essential to measure the effectiveness of rehabilitation efforts. It is crucial to note any recession and assess its stability over time, in addition to examining any concurrent mucosal infections and related esthetic problems. All participants underwent a thorough evaluation of the soft tissues surrounding their zygomatic implants using standardized ORIS indicators.

In this study, all implants were loaded immediately, usually within 48 h of placement, which was facilitated by the high primary stability obtained in all cases. Final insertion torque exceeded 45 Ncm for most implants, with only four of one patient scoring lower values (30–35 Ncm). The high torques required a cautious approach during insertion, often involving partial retrieval followed by controlled advancement to prevent excessive forces and consequent bone fracture.

This remarkable stability can be attributed to several factors, such as the tapered design at the implant apex and the minimally invasive ZAGA osteotomy technique employed for implant bed preparation. In addition, precise drilling based on anatomical considerations, underpreparation of implant trajectories, and using only one osteotomy instead of two osteotomies, as in Slot or OST, further optimized primary stability. As a result, all patients were eligible to receive immediate fixed provisional prostheses, leading to a notable reduction in the duration necessary for functional and aesthetic rehabilitation when compared to conventional bone grafting techniques.

As evidenced by this study, immediate implant loading proves to be a beneficial treatment approach, providing patients with prompt restoration of function, aesthetics, and social confidence.

This observational study also addressed patients with severe and extremely severe bone resorption, necessitating the placement of quadruple zygomatic implants. The use of narrow implants such as those described in this work facilitates the placement of four implants in narrow zygomatic bones by reducing the diameter of the osteotomy required, thereby reducing the intraoperative risk of structure invasion and zygoma fracture.

Controlled clinical studies rigorously select participants according to strict inclusion and exclusion criteria, potentially deviating from the typical patient demographics seen in routine dental settings [29]. For instance, our study did not exclude patients based on factors such as bony discontinuities in the sinus floor or nose, tobacco usage, or sinus conditions beyond acute infections. Significantly, 75% of the patients undergoing treatment presented with either partial or complete sinus occupancy, 30% were categorized as heavy smokers, and 20% showed bony defects or discontinuities at sinus or nasal levels. Hence, it is argued that results derived from clinical studies might not accurately reflect outcomes across a wider population, highlighting the importance of further observational research [30, 31]. Observational studies allow for greater treatment flexibility, yet systematic documentation of device usage and clinical outcomes provides a thorough assessment of device effectiveness [31].

## Conclusion

Despite the constraints posed by patient numbers in a non-interventional study, our findings indicate that rehabilitating the atrophic maxilla yielded a meager complication rate. Minimal changes in the frequency of late complications with respect to the one-year follow-up were observed. This was particularly evident in cases presenting extreme anatomical challenges, which were included in this study. Notably, the implant and prosthesis survival rate was 100% throughout the follow-up period of 38 to 53 months (with a mean of 46.5 months). These results underscore the effectiveness of utilizing ZAGA-Flat and ZAGA-Round site-specific zygomatic implants following the ZAGA concept as a promising treatment option for addressing severe maxillary resorption.

## Data Availability

The data sets generated and analyzed during the present study are available from the corresponding author upon reasonable request.

## Abbreviations

AS: Eng. Aleix Solà
B Al-N: Prof. Bilal Al-Nawas
CA: Dr. Carlos Aparicio
CBCT: Cone-beam computed tomography
ENT: Ear, nose, throat
FL: Fatigue limit
FOV: Field of view
L–K: Lanza Kennedy task force for rhino-sinusitis clinical diagnosis
L–M: Lund Mackay score
M L–M: Modified Lund Mackay score
OST: Original surgical technique
ORIS: Prosthetic offset, rhinosinus status, soft tissue infection/inflammation, stability
P–C: Distance from the palate half-way point to the crest midpoint distances
P–I: Distance from the palate halfway point to implant head center
UTS: Ultimate tensile strength
ZAGA: Zygoma anatomy-guided approach
ZI: Zygomatic implants
